# The neuronal potassium current I_A_ is a potential target for pain during chronic inflammation

**DOI:** 10.14814/phy2.14975

**Published:** 2021-08-17

**Authors:** Michael Biet, Marc‐André Dansereau, Philippe Sarret, Robert Dumaine

**Affiliations:** ^1^ Département de Pharmacologie et Physiologie Institut de pharmacologie de Sherbrooke Centre de Recherche du Centre Hospitalier Universitaire de Sherbrooke Faculté de médecine et des Sciences de la Santé Université de Sherbrooke Sherbrooke Québec Canada

**Keywords:** chronic inflammation, dorsal root ganglia, electrohysiology, pain

## Abstract

Voltage‐gated ion channels play a key role in the action potential (AP) initiation and its propagation in sensory neurons. Modulation of their activity during chronic inflammation creates a persistent pain state. In this study, we sought to determine how peripheral inflammation caused by complete Freund's adjuvant (CFA) alters the fast sodium (I_Na_), L‐type calcium (I_CaL_), and potassium (I_K_) currents in primary afferent fibers to increase nociception. In our model, intraplantar administration of CFA induced mechanical allodynia and thermal hyperalgesia at day 14 post‐injection. Using whole‐cell patch‐clamp recording in dissociated small (C), medium (Aδ), and large‐sized (Aβ) rat dorsal root ganglion (DRG) neurons, we found that CFA prolonged the AP duration and increased the amplitude of the tetrodotoxin‐resistant (TTX‐r) I_Na_ in Aβ fibers. In addition, CFA accelerated the recovery of I_Na_ from inactivation in C and Aδ nociceptive fibers but enhanced the late sodium current (I_NaL_) only in Aδ and Aβ neurons. Inflammation similarly reduced the amplitude of I_CaL_ in each neuronal cell type. Fourteen days after injection, CFA reduced both components of I_K_ (I_Kdr_ and I_A_) in Aδ fibers. We also found that I_A_ was significantly larger in C and Aδ neurons in normal conditions and during chronic inflammation. Our data, therefore, suggest that targeting the transient potassium current I_A_ represents an efficient way to shift the balance toward antinociception during inflammation, since its activation will selectively decrease the AP duration in nociceptive fibers. Altogether, our data indicate that complex interactions between I_K_, I_Na_, and I_CaL_ reduce pain threshold by concomitantly enhancing the activity of nociceptive neurons and reducing the inhibitory action of Aβ fibers during chronic inflammation.

## Graphical abstract

Inflammatory pain linked to the differential expression of potassium currents within the dorsal root ganglia.

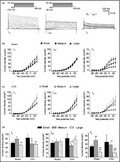

**Keypoint summary**

- Pain during chronic inflammation is linked to the alteration of the electrical properties of at least three types of dorsal root ganglion (DRG) neurons. However, our understanding of pain signaling is incomplete due to our lack of knowledge of how these three cell types electrically interact.
- Using the patch‐clamp technique, we characterized the effects of chronic inflammation caused by complete Freund's adjuvant (CFA) on the action potential (AP) and ionic currents: I_Na_ (sodium), I_CaL_ (calcium), and I_K_ (potassium) in these three afferent neuronal cell types isolated from rat DRGs.
- To our surprise, I_A_—the fast component of I_K_—was poorly expressed in large proprioceptive neurons but abundant in nociceptive medium‐ and small‐sized neurons.
- Our study, therefore, revealed a previously unknown distribution of I_Ka_ currents within three types of DRG neurons. Such specific expression of I_A_ may provide a basis to establish new pain treatment paradigms targeting more specifically nociceptive neurons.

## 1 INTRODUCTION

Pain sensation typically originates in primary sensory neurons known as nociceptors, which have their cell bodies located within the peripheral dorsal root ganglia (DRG; Dubin & Patapoutian, 2010). These nociceptors innervate target tissues, such as skin, joints, muscles, and visceral organs. They respond to thermal, mechanical or chemical noxious stimuli and convey the sensory information to the spinal dorsal horn and from there to upper brain structures. According to the size of their diameter, these excitatory neurons can be subdivided into three main classes (Crawford & Caterina, 2020; Rambourg et al., 1983). Small unmyelinated C and lightly myelinated medium‐sized Aδ fibers are primarily involved in nociceptive processing. In contrast, large non‐nociceptive DRG neurons (e.g., heavily myelinated Aβ fibers) are conducting proprioception and mechanoreception and normally dampen the pain signal in the spinal cord through the activation of inhibitory interneurons (Basbaum et al., 2009; Guo & Hu, 2014).

Neuronal activity within DRGs depends critically on the firing frequency of action potentials (AP) of each neuronal cell type. Parameters, such as the AP threshold, its amplitude, and duration (APD), as well as the refractory period are modulators of firing frequency and excitability of sensory neurons. The characteristics and propagation of APs along the axons and the processing of the nociceptive signal rely critically on the electrical activity of ion channels expressed in primary afferent neurons (Waxman & Zamponi, 2014). Among them, voltage‐gated ion channels are the key regulators of sensory neuron excitability (Linley et al., 2010) and AP firing frequency. Indeed, pain sensation during acute and chronic inflammation is intimately coupled with the release of a wide range of molecular mediators that alter the activity of voltage‐gated ion channels to either sensitize or directly excite peripheral sensory nerve terminals (Matsuda et al., 2019; Pinho‐Ribeiro et al., 2017). The resulting neuronal hyperexcitability generated by these mediators contributes to the development of exaggerated pain sensation, such as hyperalgesia and spontaneous pain that can lead to peripheral sensitization and then translated into a chronic pain state (Pace et al., 2018).

The nociceptive information transmitted by sensory neurons thus depends on a fine balance between inward and outward electrical currents. We and others have previously shown that the hyperexcitability of sensory neurons after peripheral inflammation is linked to the modulation of the sodium current I_Na_ and expression of tetrodotoxin TTX‐resistant channels (Belkouch et al., 2014; Bennett et al., 2019; Black et al., 2004; Tanaka et al., 1998). Peripheral tissue injury also enhances the activity of voltage‐gated L type calcium I_CaL_ and the potassium current I_K_ that participate in neuronal depolarization and repolarization, respectively (Abdulla & Smith, 2001; Everill & Kocsis, 1999; Ishikawa et al., 1999; Yang et al., 2004). In neurons, a rapidly inactivating 4‐AP‐sensitive A‐type K^+^ current (I_Ka_) and a sustained delayed TEA‐sensitive K^+^ current (I_Kdr_) contribute to generate I_K_. These electrical currents are strong modulators of APD, excitability and conduction, and their functional cooperation regulates the neuronal firing rate and as a correlate, each neuronal cell type expresses a unique set of voltage‐gated channels.

Abnormal excitability of primary sensory neurons is attributable to alterations in the expression and functional characteristics of those voltage‐gated ion channels. However, most studies report the effects of neuronal injuries only in one neuronal subtype and, in most cases, focus on a particular electrical current. As a consequence, our global understanding of the electrophysiological mechanisms controlling the interaction between nociceptive and non‐nociceptive neurons within DRGs remains incomplete. In the present study, we thus compared the activity of I_Na_, I_K,_ and I_Ca_ in small, medium, and large‐sized neurons from lumbar DRGs. Our goal was to identify how chronic inflammation caused by complete Freund's adjuvant (CFA) differentially modulated their electrophysiological properties and changed the AP morphology to tip the balance between nociceptive and non‐nociceptive signals.

## 2 METHODS

### 2.1 Ethical approval

All protocols for euthanasia and care of the animals followed the Canadian Council on Animal Care (CCAC) and were approved by the University of Sherbrooke Animal Ethical Care Committee and follows the ARRIVE guidelines as requested in the principles and standards for reporting animal experiments from the Journal of Physiology and Experimental Physiology journals. Each author is aware and understands the ethical principles under which the journal operates and that their work complies with the animal ethics checklist as outlined in the editorial guidelines of the Journal of Physiology.

### 2.2 Chronic inflammatory pain

All experimental procedures were approved by the Animal Care and Use Committee of the Université de Sherbrooke and were in accordance with the policies and directives of the Canadian Council on Animal Care and guidelines from the International Association for the Study of Pain (IASP). Adult male Sprague–Dawley rats (200–225 g, Charles River) were housed two per cage in a climate‐controlled room on a 12 h light/dark cycle with free access to food and tap water. Complete Freund's adjuvant (CFA, Calbiochem) was prepared with 7 mg/ml of *Mycobacterium butyricum* (Difco Laboratories) and emulsified 1:1 with saline 0.9%. Rats under light anesthesia with isoflurane received an intraplantar injection of 100 μl (400 μg) of the freshly emulsified CFA mixture into the left hind paw. Sham animals received an intraplantar injection of 100 μl of saline.

### 2.3 Behavioral studies

Mechanical hypersensitivity was assessed using von Frey hairs, while thermal hypersensitivity was determined using a plantar test analgesia meter emitting heat through a focus I.R. light aimed at the hind paw of the animal. Both mechanical allodynia and thermal hyperalgesia were recorded on the day before CFA administration and on days 3, 7, and 14 following CFA administration. Animals were allowed 7 days to habituate to the animal facility. They were then habituated for 5 min daily for three consecutive days in each holding cage, sequentially. First, they were put in a plastic cage on an elevated mesh bottom allowing access to the paws for the von Frey test. Then, they were put on a plastic cage resting on a glass pane that allows heat transmission. For the von Frey test, the trial was initiated with stimulation with a 2 g filament. Stimuli were then presented consecutively: descending following a positive response, ascending otherwise. Stimulation was performed four times following the first positive response and was stopped after a negative response to the biggest hair used (15 g). The 50% threshold was calculated using the formula: 50% g threshold = 10^(Xf+kδ)^, where Xf = value (in log units) of the final von Frey hair used; k = tabular value for the pattern of positive/negative responses; and γ = mean difference (in log units) between the different stimuli (here, 0.2). The range of stimuli used was: 0.4, 0.6, 1, 1.4, 2, 4, 6, 10, and 15 g.

Immediately after undergoing the von Frey test, rats were transferred to the plantar test analgesia meter apparatus. A radiant noxious heat source was then applied through the glass onto the mid‐plantar surface of the hind paw. The time elapsed between the onset of the stimulus and manifestation of the paw withdrawal response was automatically recorded and considered as an index of the heat nociceptive threshold. The intensity of the mobile infrared heat source was adjusted to 60 Hz. A cutoff time of 30 s was imposed to prevent tissue damage. Their hind paw was stimulated three times at 5 min intervals and the three measurements of latency were averaged for each animal.

The hind paw edema was evaluated using a digital plethysmometer, by measuring water displacement produced by the immersion of the animal paw in one of the two interconnected tubes. This induced a change in conductance between the two platinum electrodes in the second tube.

### 2.4 Statistical analysis of behavioral data

Data were analyzed using a two‐way ANOVA and a Sidak correction in order to compare CFA‐treated rats with Sham animals at different time points. *N* = 6 animals for each condition (for a total of 12 animals). For the von Frey test, *F*_treatment_(1,10) = 41,48. For the plantar test, *F*_treatment_(1,10) = 6,119. For the edema, *F*_treatment_(1,10) = 182,3. All data passed the Shapiro–Wilk normality test.

### 2.5 Preparation of dorsal root ganglion (DRG) neurons

Fourteen days after CFA administration, rats were euthanized by decapitation in accordance with the CCAC and European Directive 2010/63/EU. This procedure was necessary to avoid anesthesia‐induced artifacts on measurements of ionic currents. Cells were dissociated from lumbar (L4–L6) dorsal root ganglia of adult rats and kept in primary culture for a maximum period of 20 h, as previously described (Belkouch et al., 2014). Briefly, DRGs isolated from Sham and CFA‐injected rats were dissociated from their adherent connective tissues. After washing with calcium‐magnesium free PBS (pH 7.4), the DRGs were sequentially incubated for 1h30 in a 0.25% collagenase A solution (0.505 U/ml; Roche Diagnostics) and for 30 min in 3 ml of trypsin (0.25%; GIBCO). Ganglia were then mechanically dissociated into single cells by trituration with polished Pasteur pipettes in the culture medium containing 1:1 Dulbecco's modified Eagle's medium (DMEM, Invitrogen) and Ham's F12 supplemented with 10% fetal bovine serum (GIBCO) and 1% penicillin (100 U/ml)/streptomycin (0.1 mg/ml). Finally, isolated DRG neurons were plated onto poly‐D‐lysine/laminin‐coated glass coverslips and placed at 37℃ in a humidified incubator (5% CO_2_) prior to electrophysiological recordings. The density of the cell plating was determined empirically to obtain a sufficient number of neurons for patch‐clamp without overlapping of cells. For each animal, six dishes were prepared.

### 2.6 Electrophysiological recordings

Neurons were divided into small (<30 pF), medium (>30 pF; <70 pF), and large DRGs (>70 pF) based on the capacitance calculated from the integral of the capacitive current recorded during a 10 mV pulse in a range of voltage where no active current was triggered. Liquid junction potential was measured after breaking the tip of the electrode and found to be less than 3 mV in most experiments, as predicted by the transport numbers and concentrations of salt used. We did not compensate for such a small difference. Series resistance was always over 600 mOhms and this was one of our quality criteria. Cells displaying leaks were discarded. In some instances, small changes during the experiments were compensated using the leak subtraction procedures on the Axon amplifier. Cells that could not be fully compensated or displayed anodal breakdown during the measurement of I_Na_ were discarded.

Following the formation of a giga‐seal, the cell membrane was ruptured and maintained under voltage clamp with whole‐cell recording configuration. Series resistance and capacitance were compensated before switching to the current clamp. Resting membrane potentials were measured as the membrane voltage when I = 0 and series resistance was compensated to 85%. Afterward, cells were held at −60 mV by injecting a constant current between 50 pA and 150 pA. We choose this voltage based on cell resting membrane potential (RMP) measurements between −55 mV and −60 mV in each cell type (Table 1; Figure S1). This enabled us to standardize action potential (AP) measurements. Single (non‐spontaneous) action potentials were measured in current‐clamp mode and triggered by a 1 ms current stimulus step between 20 and 600 pA in increments of 20 pA (Figure S1). Cells were superfused with a solution containing (in mM): 126 NaCl, 5.4 KCl, 2.0 CaCl_2_, 1.0 MgCl_2_, 20 HEPES, and 11 glucose (pH 7.4 with NaOH). The minimum current needed to evoke a full action potential with an overshoot was used as the threshold current. Although in some cases a 1 ms test pulse was used, the voltage step generated at the threshold was well below the maximum amplitude and within the rising phase of the AP. Small artifactual voltage ramps were sometimes observed at the foot of the action potential but did not interfere with our V_max_ or APD measurements. Pipette solution contained in mM: 90 K‐aspartate, 30 KCl, 5 NaCl, 1.0 MgCl_2_, 6 EGTA, 10 HEPES, 5.5 glucose, and 4 Na_2_‐ATP (pH 7.2 with KOH).

**TABLE 1:** Action potential parameters during spontaneous activity. RMP: Resting membrane potential of the cells after the establishment of giga‐seal. APA: Action potential amplitude, OS: overshoot, I_Th_: threshold current. N: number of cells tested. Data ± SEM from four animals. Statistical significance: *p < 0.05, ***p < 0.01 compared to Sham

		RMP (mV)	APA (mV)	AHP (mV)	OS (mV)	I_Th_ (pA)	*n*
SHAM	Small	−56 ± 3	99 ± 3	−70 ± 2	37 ± 3	280 ± 20	5
	Medium	−59 ± 2	101 ± 4	−69 ± 2	41 ± 4	183 ± 54	6
	Large	−56 ± 1	115 ± 3	−67 ± 1	53± 3	583 ± 119	6
CFA	Small	−57 ± 3	97 ± 7	−59 ± 4*	34 ± 7	150 ± 27***	10
	Medium	−54 ± 3	107 ± 3	−57 ± 3*	46 ± 3	250 ± 43	10
	Large	−56 ± 3	115 ± 3	−58 ± 3*	53 ± 2	190 ± 43***	10

Sodium, calcium, and potassium currents were recorded at room temperature (22℃) in the whole‐cell configuration of the patch‐clamp technique using an Axopatch 200B amplifier (Axon Instruments) as previously described (Biet et al., 2015; Ton et al., 2017).

Tetrodotoxin‐resistant (TTX‐R) sodium currents (I_Na_) known to contribute to nociception (Amaya et al., 2000; Amir et al., 2006; Belkouch et al., 2014; Dib‐Hajj et al., 2010; Gold et al., 1996) were recorded by adding 100 nM of the sodium channel blocker tetrodotoxin (TTX) in a solution containing (in mM): 125 NaCl, 5 NaOH, 2.8 Na acetate, 4 KOH, 0.5 CaCl_2_, 1.5 MgCl_2_, 20 HEPES, and 10 glucose (pH 7.4 with NaOH). For I_Na_ measurements, tetraethylammonium (5 mM), BaCl2 (5 mM), and CoCl2 (1 mM) were added to inhibit endogenous K^+^ and Ca^2+^ currents, respectively. The pipette solution contained (in mM): 15 NaCl, 5 KCl, 120 CsCl, 5 KCl, 1.0 MgCl_2_, 4 Na_2_‐ATP, 10 EGTA, and 10 HEPES (pH 7.3 with CsOH). Whole‐cell capacitance and series resistance compensation (85%) were optimized to minimize the duration of the capacitive artifact and reduce voltage errors. I_Na_ was elicited by a series of 60 ms depolarizing steps between −60 and +60 in 5 mV increments from a holding potential of −80 mV. Standard inactivation protocol consisted in a series of 500‐ms inactivating pulses from −70 to +20 mV in an increment of 5 mV from a holding potential of −80 mV followed by a 15 ms test pulse to +10 mV to open all available channels. Late sodium current (I_NaL_) was measured at the end of the 60 ms used to measure I_Na_. To measure recovery from inactivation, cells were maintained at −100 mV and I_Na_ was elicited by two 40 ms pulses to 10 mV separated by progressively longer periods of rest to the holding potential.

For calcium current measurements (I_Ca_), neurons were superfused with a solution containing (in mM): 140 TEA‐Cl, 5 CaCl_2_, 2 MgCl_2_, 10 HEPES, and 10 glucose (pH 7.4 with CsOH). The pipette solution contained (in mM): 100 CsCl, 20 TEA‐Cl, 10 EGTA, 10 HEPES, 5 Na_2_‐ATP, and 0.4 Na_2_‐GTP (pH 7.2 with CsOH). I_Ca_ was elicited by a series of 600 ms depolarizing steps between −50 and +60 mV in 5 mV increments from a holding potential of −60 mV.

The rundown of I_CaL_ neurons occurs much more slowly in neurons when the intracellular solution contains EGTA. While BaCl2 is often used to characterize the biophysics of calcium channels, we opted to not use barium. This may yield smaller currents due to rundown but we deliberately choose this compromise since this barium interferes with the natural inactivation of I_Ca_ and this would prevent us to make a realistic correlation with the effects of I_Ca_ on the action potential duration. In our experiments, we were able to maintain a stable I_Ca_ for the duration of the experiment which lasts between 15 and 20 min. Cells displaying unstable currents within this time frame were discarded.

Potassium currents (I_K_) were measured in cells bathed in an external solution containing (in mM): 130 Choline‐Cl, 5 KOH, 2 MgCl_2_, 2 CaCl_2_, 10 HEPES, and 12 glucose (pH 7.4 with HCl). Pipette solution contained (in mM): 120 K‐Asp, 20 NMDG, 11 EGTA, 10 HEPES, 2.5 Mg‐ATP, and 0.5 LiCl (pH 7.2 with NMDG). Total I_K_ was elicited by a conditioning pre‐pulse of 1s at −100 mV followed by a series of 500 ms depolarizing steps between −80 and +80 mV in 10 mV increments from a holding potential of −60 mV. I_K_ is composed of two potassium currents, I_Kdr_ and I_A_. The delayed rectifier current I_Kdr_ was elicited by a series of 500 ms depolarizing steps between −80 and +80 mV in 10 mV increments preceded by a conditioning pulse of 1s at −30 mV to inactivate the A‐type current I_A_. Holding potential was set at −60 mV. I_A_ was obtained by subtracting I_Kdr_ from I_K_.

All solutions were adjusted at 300 mOsm with sucrose. Pipettes were pulled from Corning 7052 glass (Model PP‐89, Narashige) and had resistance between 1.5 and 3 MΩ. Currents were filtered at 5 kHz and digitized at 10–50 kHz with a model 1444 Digidata from Axon Instruments.

### 2.7 Data analysis

For the clarity of the figures, normalized data are expressed as ±SEM (standard deviation). Current data acquisition and analysis were performed using the pCLAMP program suite V9.2 (Axon Instruments), EXCEL (Microsoft), and ORIGIN 8 (Microcal Software) software, respectively. Activation and inactivation data were fitted to a standard Boltzmann distribution function: $Y=\frac{\left(A^{1}‐A^{2}\right)}{1‐\exp\left[\left(V^{m}‐E\right)/V^{0.5}\right]}+A^{2}$ where Y represents the fraction of activated (m) or available (h) channels obtained, respectively, from the ratio of the macroscopic conductance (G_Na_/G_Na,Max_) or the sodium current I/I_Max_. V_m_: membrane test potential, E: sodium current reversal potential, and V_0.5_ is the mid‐potential for activation or inactivation. G_Na_ was obtained from the current–voltage relationship as G_Na_ = I_Na_/(V_m_ − E) and G_Na, Max_ represents the maximal Na^+^ conductance (slope of the linear portion of the I/V relationship).

### 2.8 Statistical analysis of electrophysiological data

Statistical analyses were performed using the Microsoft Excel and Origin v8, MicroCal statistical packages. Differences between groups were assessed using the nonparametric Mann–Whitney test.

## 3 RESULTS

**FIGURE 1:**
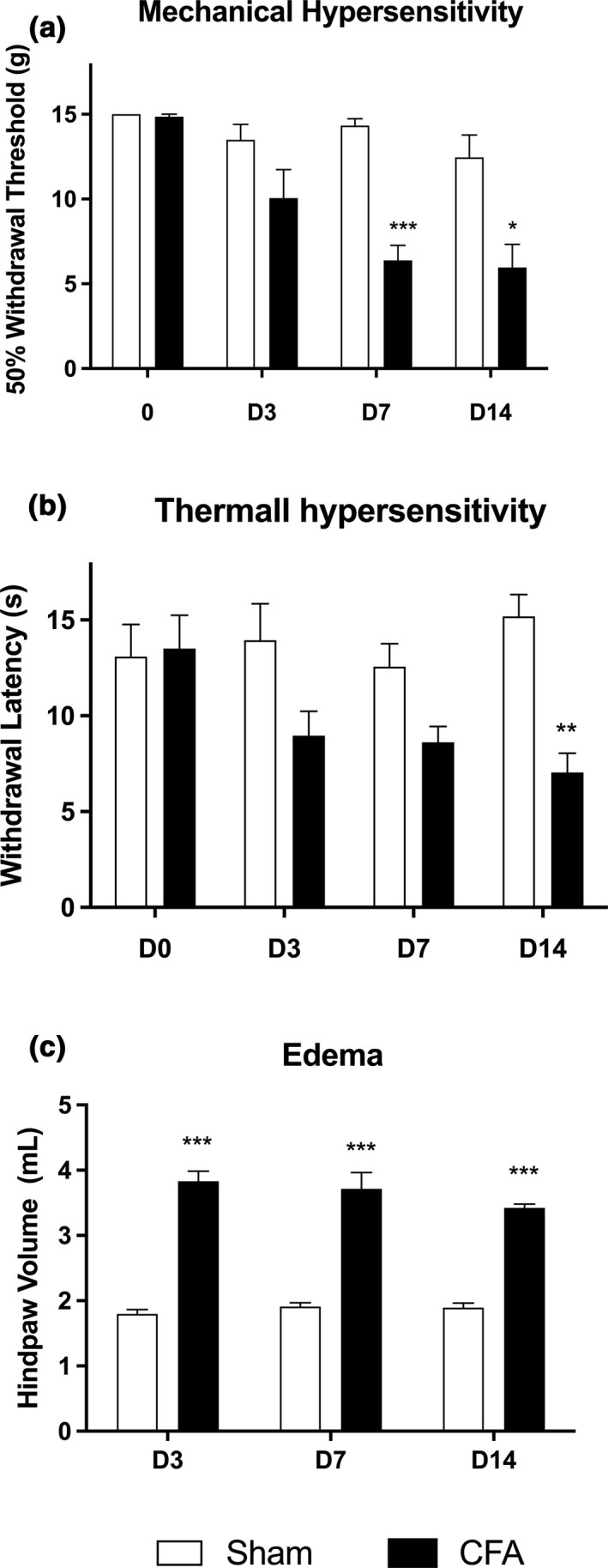
Effect of complete Freund's adjuvant on pain perception. Intraplantar injection of CFA induces mechanical hypersensitivity (a) and thermal hypersensitivity (b) that manifest early and are well‐established by day 14. In addition, severe edema is observed on the ipsilateral hind paw (c). *p < 0.05. **p < 0.01. ***p < 0.001 compared to the Sham group at the same time point. *N* = 6 animals per condition (CFA/Sham)

The time course of two behavioral endpoints to punctate mechanical and heat stimuli was examined over 14 days following the unilateral injection of complete Freund's adjuvant (CFA) into the plantar surface of the rat's hind paw. As shown in Figure 1, CFA‐treated rats developed both tactile allodynia and thermal hyperalgesia. Mechanical hypersensitivity was evidenced on day 3 post‐injection by a reduced paw withdrawal threshold compared to baseline values or Sham animals and was maintained until day 14 (Figure 1a). Concomitantly, CFA‐injected animals exhibited a decreased paw withdrawal latency of the ipsilateral hind paw in response to a noxious radiant heat stimulus (Figure 1b). The inflammation primed by CFA also resulted in a progressive swelling, leading to ipsilateral paw edema (Figure 1c).

We next determined how CFA‐induced inflammation influenced the morphology of the action potential (AP) in the three main subsets of neurons present in primary cultures from dissected rat DRGs. Sensory neurons were separated on the basis of cell size into small, medium, and large cells according to their capacitance, measured from patch‐clamp recordings. Table 1 shows the parameters from action potential recorded immediately after the establishment of a giga‐seal. We found that CFA did not increase the amplitude of the action potentials, suggesting that chronic inflammation may have a limited impact on the amplitude of the sodium current. The AP hyperpolarized tail of the action potential (AHP) was also significantly reduced in inflammation, indicating potential changes in the activity of repolarizing potassium currents. We also observed that less current was needed in CFA animals to trigger spontaneous action potentials.

The average resting membrane potential in cells from both Sham and CFA animals was in the vicinity of −58 mV. In order to avoid recording artifacts linked to differences in resting membrane potentials and inactivation of sodium channels, we used a holding potential of −60 mV in all subsequent experiments.

**FIGURE 2:**
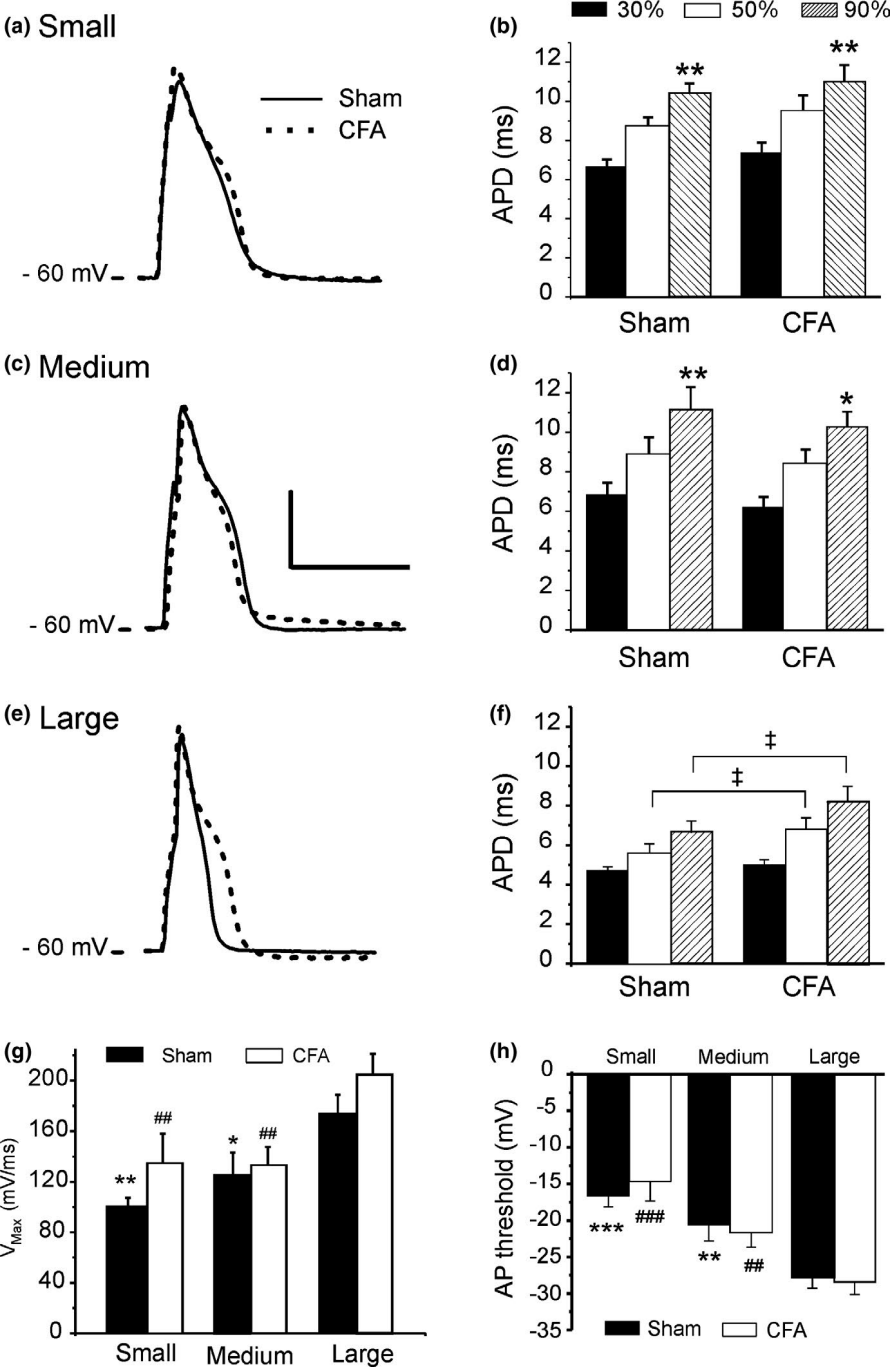
CFA induces the prolongation of action potential duration (APD) in large neurons but not in small and medium‐sized cells Data ± SEM. (a, c, e) Representative action potential recordings from each neuronal cell type. Scale bar: 35 mV/15 ms (b, d, f) APD at 30%, 50%, and 90% of repolarization (relative voltage drop from the peak of the AP) for each cell type. Statistical significance (ANOVA): *: comparison to APD30 within each condition *p < 0.05, **p < 0.01. ^‡^ *p* < 0.01 CFA versus Sham (g) Maximum rate of depolarization (V_max_) during the initial phase 0 of the AP. Statistical significance: *p < 0.05, **p < 0.01 versus large neurons (Sham condition), ^##^ *p *< 0.01 versus large cells (CFA condition). (h) AP threshold in each neuronal cell type. Statistical significance: **p < 0.01, ***p < 0.001 versus large neurons (Sham condition), ^##^ *p *< 0.01, ^###^ *p *< 0.001 versus large cells (CFA condition). Number of cells for each cell type: 13 (Sham) and 15 (CFA) from four rats in each condition

Figure 2 shows that the action potential duration (APD) was longer in small and medium‐sized neurons compared to large cells (Figure 2a–f). AP in small and medium‐sized neurons displayed a marked inflection point (plateau) around 30% repolarization, suggesting the contribution of a large inward current during the initial phase of repolarization. In large neurons, no plateau phase was observed and repolarization rate remained similar before or after 30% repolarization (Figure 2e,f), thus suggesting a balance of outward and inward currents favoring early repolarization. Inflammation did not influence APD in small and medium‐sized neurons. However, CFA prolonged the APD in large cells and a strong inflection point appeared after 30% repolarization (Figure 2e), indicative of an increase in inward calcium or late sodium currents contributing to the early phase of repolarization. The maximum rate of depolarization was highest in large neurons, indicating a larger fast sodium current (I_Na_), but no difference was observed between small and medium neurons (Figure 2g). However, inflammation did not influence the maximum rate of depolarization (V_max_) in any cell type. In addition, the threshold voltage for triggering an action potential was 12 mV and 8 mV more positive in small and medium cells, respectively, compared to large neurons. This result suggests that large neurons have a smaller voltage gap between their normal resting membrane potential and AP threshold and are more readily excitable (Figure 2h). The AP voltage threshold (Figure 2h) was not affected by CFA in any of the three types of neurons and therefore did not directly influence cell excitability despite a reduction in current needed to reach the threshold (Table 1). This suggests that CFA may alter ionic currents to increase electrical membrane resistance at rest such that a less depolarizing current is needed to reach the voltage threshold. Overall, the most prominent effect of CFA was to reduce the difference in APD between large and small / medium‐sized neurons.

APD is mostly determined by the balance of currents between the outward potassium current (I_K_), the inward calcium current (I_Ca_), and the sustained (late) component of the sodium current (I_NaL_). We next sought to determine how CFA altered this balance of currents to prolong APD.

### 3.1 Effects on IK

**FIGURE 3:**
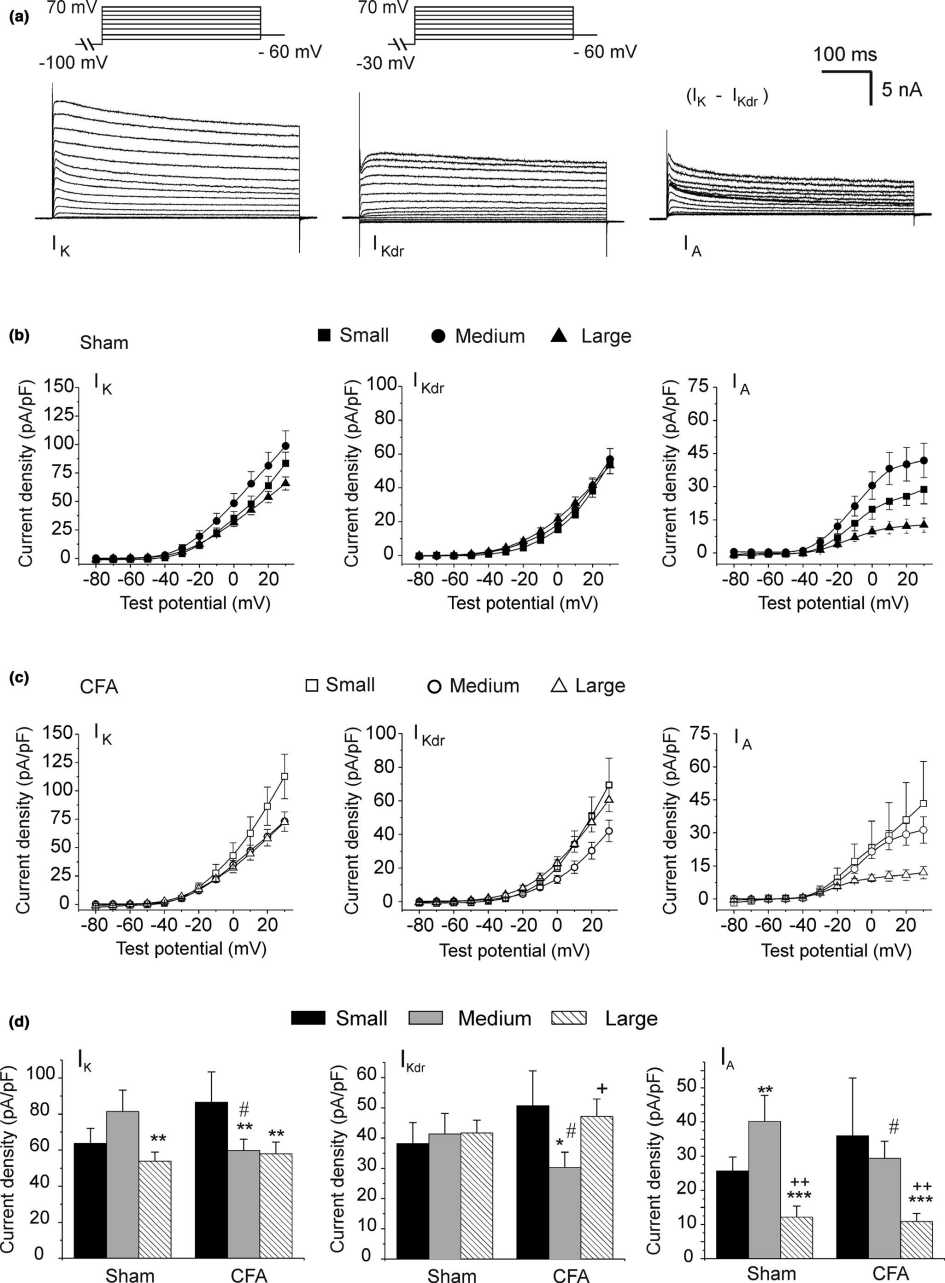
Inflammation selectively reduces potassium currents in medium neurons. (a): Representative current recordings of total (I_K_), delayed rectifier (I_Kdr_), and A‐type (I_A_) potassium currents in medium DRG neurons. A 1s conditioning step (truncated in the inset) to −30 mV was used to inactivate I_A_ and record I_Kdr_ (see Section 2). The holding membrane potential of the cells was −60 mV. The transient outward current I_A_ was obtained by the digital subtraction of I_Kdr_ current traces from I_K_. (b and c) Current–voltage relationship for each current in Sham and CFA animals. Currents normalized to cell capacitance indicate current density for each step potential. All data are expressed with SEM for clarity of the figures. (d) Current density at +20 mV. Statistical significance: *p < 0.05, **p < 0.01, ***p < 0.001 (vs. small neurons); ^+^ *p*<0.05, ^++^ *p *< 0.01 (vs. mid cells); ^#^ *p *< 0.05 versus Sham. Number of cells for Sham and CFA conditions, respectively: small: 10 and 8; medium: 12 and 11; large: 13 and 9; from 4 Sham and 4 CFA‐treated animals

Since large neurons displayed a longer APD in CFA condition, we initially focused on potassium currents involved in neuronal repolarization. As previously described (Furuta et al., 2012), rat DRG neurons harbor at least two types of voltage‐dependent potassium currents, a delayed rectifier current (I_Kdr_) and a type A transient outward current (I_A_). Consistent with these findings, we measured total potassium current (I_K_) with biphasic inactivation kinetics. To separate the two components, we first used a 1 s conditioning prepulse to −100 mV to obtain (I_K_) and a second protocol with a prepulse to −30 mV to obtain I_Kdr_. Digital subtraction of the two recordings yielded I_A_ (Figure 3a). Analysis of the current–voltage revealed that I_K_ was smaller in large neurons from Sham animals due to a reduced contribution of I_A_ (Figure 3b,d). Although we noticed a trend for a larger I_K_ in medium Sham neurons (I/V relationship), the difference did not reach significance levels in our experiments even when measured at +20 mV (Figure 3d). This trend in Sham neurons seemed to stem from a larger I_A_ in medium‐sized cells (Figure 3d). We did not observe changes in I_K_ in small and large cells from CFA‐treated animals when compared to Sham. However, CFA selectively reduced the amplitude of I_K_ in medium cells, thus eliminating the K^+^ current gradient between large and medium neurons. This reduction was due to a decrease in both I_Kdr_ and I_A_ amplitude. Interestingly, CFA had no effect on I_Kdr_ or I_A_ in small and large neurons. Thus, CFA specifically targeted potassium currents in medium‐sized cells.

**FIGURE 4:**
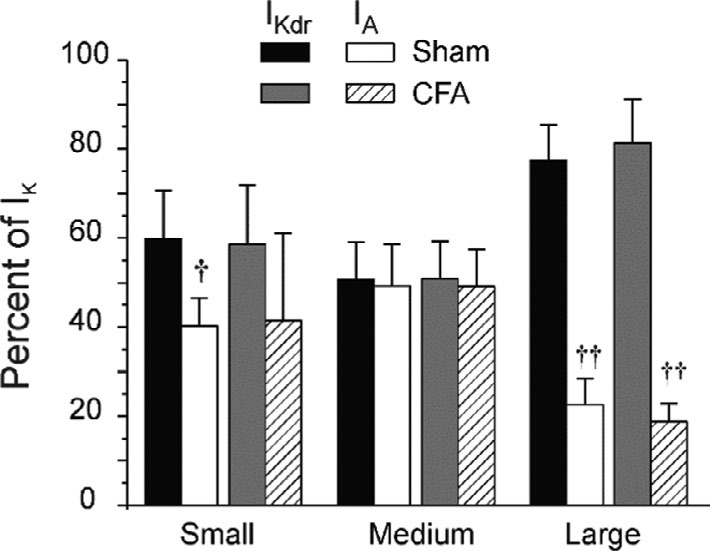
Fraction of I_Kdr_ and I_A_ contributing to I_K_ expressed as % of the total current. Significance level: ^†^ *p *< 0.05 and ^††^ *p *< 0.01 compared to I_Kdr_. Number of cells as mentioned in Figure 3. Data ± SEM

We next evaluated the relative contribution of I_Kdr_ and I_A_ to the total potassium current in all neuronal cell types. Figure 4 shows that contributions of I_Kdr_ and I_A_ to I_K_ were relatively equal (50%) in medium cells from Sham rats. In small cells, the I_A_ fraction was slightly lower accounting for roughly 40% of I_K_. Surprisingly, I_A_ accounted for only 20% of the total current in large cells and therefore contributed minimally to repolarization in large neurons. CFA had no effect on the contribution of I_Kdr_ and I_A_, thus suggesting that the reduction of I_K_ in medium‐sized neurons was not due to the differential expression of ion channels.

### 3.2 Effects on I_Na_

Neuronal excitability and conduction in DRGs are determined by the activation of the sodium current I_Na_. This current comprises a peak and a late component. Fast activation occurs within milliseconds and generates the peak current responsible for the rising phase of the action potential, conduction, and cell excitability. Activation is then followed by the inactivation of sodium channels which generates a slow (late) inward component (I_NaL_) that opposes I_K_ and modulates APD. We initially tested for potential changes in excitability during CFA‐induced inflammation by characterizing the activation of I_Na_.

**FIGURE 5:**
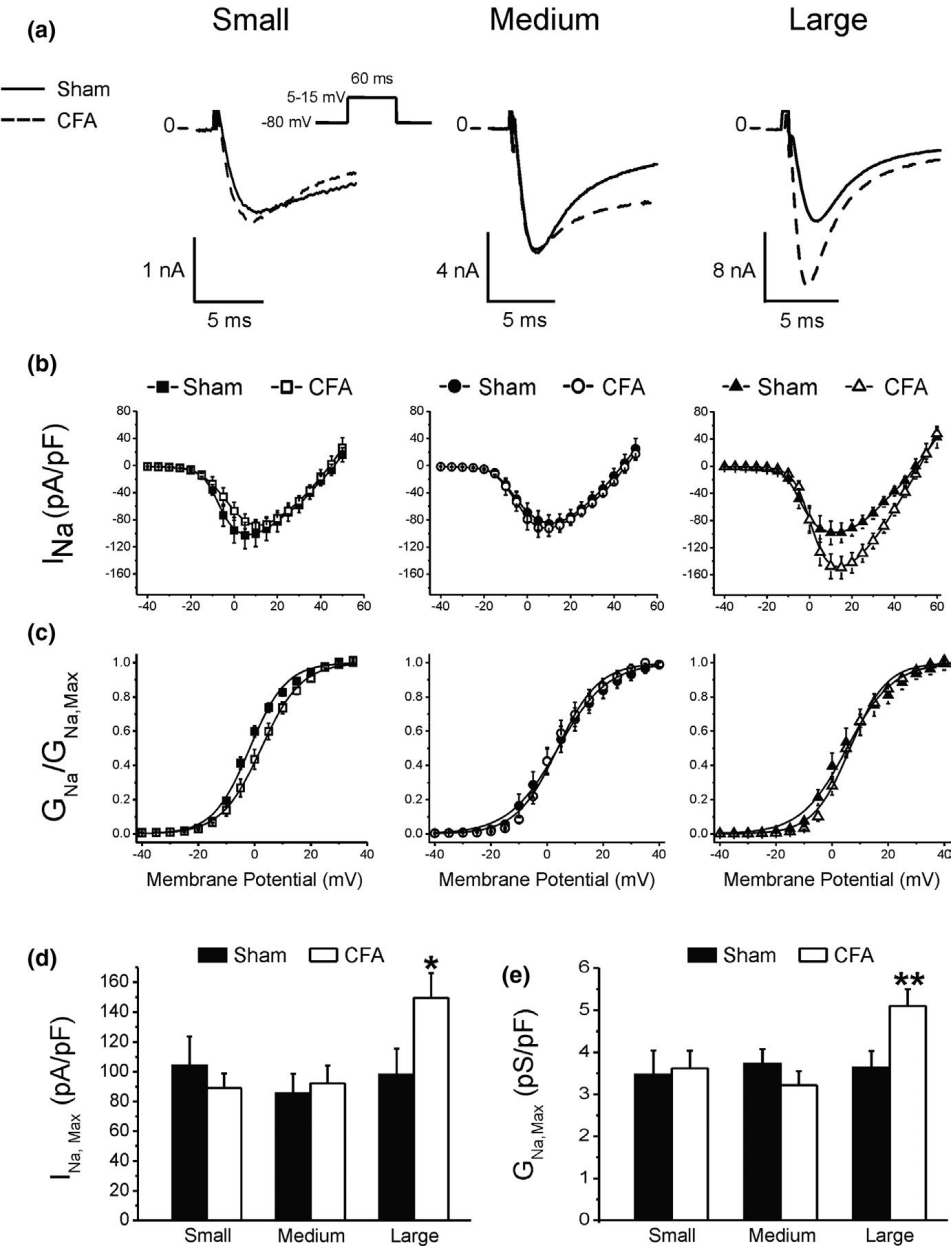
CFA selectively increases the amplitude of TTX‐r I_Na_ in large neurons. (a) Representative patch‐clamp recording of the sodium current I_Na_ in the three neuronal cell types. (b) Current–voltage relationship (I/V) in Sham and CFA‐treated animals. Individual I_Na_ was normalized to the capacitance of their respective cell and averaged to express current density. (c) Fraction of channels opened at various voltages (activation). The ratio of the conductance at each potential was normalized to the value of the maximum conductance obtained as the slope of the linear portion of the I/V relationship (b). Data ± SEM was fit to a standard Boltzmann distribution function and conductance (G_Na_) was calculated as the ratio I_Na_/(V_m_ − E_Na_), as explained in Methods. (d) Maximum current density in each neuronal cell type. (e) Maximum conductance. Statistics: *p < 0.05 versus Sham, same cell type. Number of cells: 12–13 for each cell type from six rats. In (d and e), data ±SD was plotted to illustrate the maximum spread of the data

We previously reported that CFA induced overexpression of the tetrodotoxin‐resistant (TTX‐r) sodium channel NaV1.8 in large sensory neurons (Belkouch et al., 2014). We, therefore, tested if CFA‐induced inflammation increased I_Na_ in other sensory neurons. Figure 5a–d shows that TTX‐r I_Na_ amplitude was similar in all cell types in Sham animals. CFA selectively increased peak I_Na_ in large neurons but had no effect in small and medium DRG neurons. Analysis of the current–voltage relationship (I/V, Figure 5b) revealed that CFA shifted the voltage where I_Na_ was maximum from 5 to 10 mV in small neurons but there was no significant difference in medium and large cells. These changes in I_Na_ activation in small and large neurons may in part explain the lower threshold observed in CFA‐treated animals (Figure 3g).

Mid‐activation potentials (V_0.5_) in Sham animals were −1.7 ± 0.3, 3.9 ± 0.4, and 5.3 ± 0.5 mV in small, medium, and large neurons, respectively. There was a small but significant difference (*p *< 0.05, *F*‐test) in mid‐activation potential between small and large neurons in Sham animals. CFA had no significant effect in medium and large neurons with V_0.5_ values of 3.8 ± 0.4 mV versus 6.4 ± 0.5 mV, respectively (Figure 5c) but depolarized V_0.5_ to 2.4 ± 0.2 mV in small neurons. Inflammation abolished the difference between small and large neurons observed in Sham animals.

**FIGURE 6:**
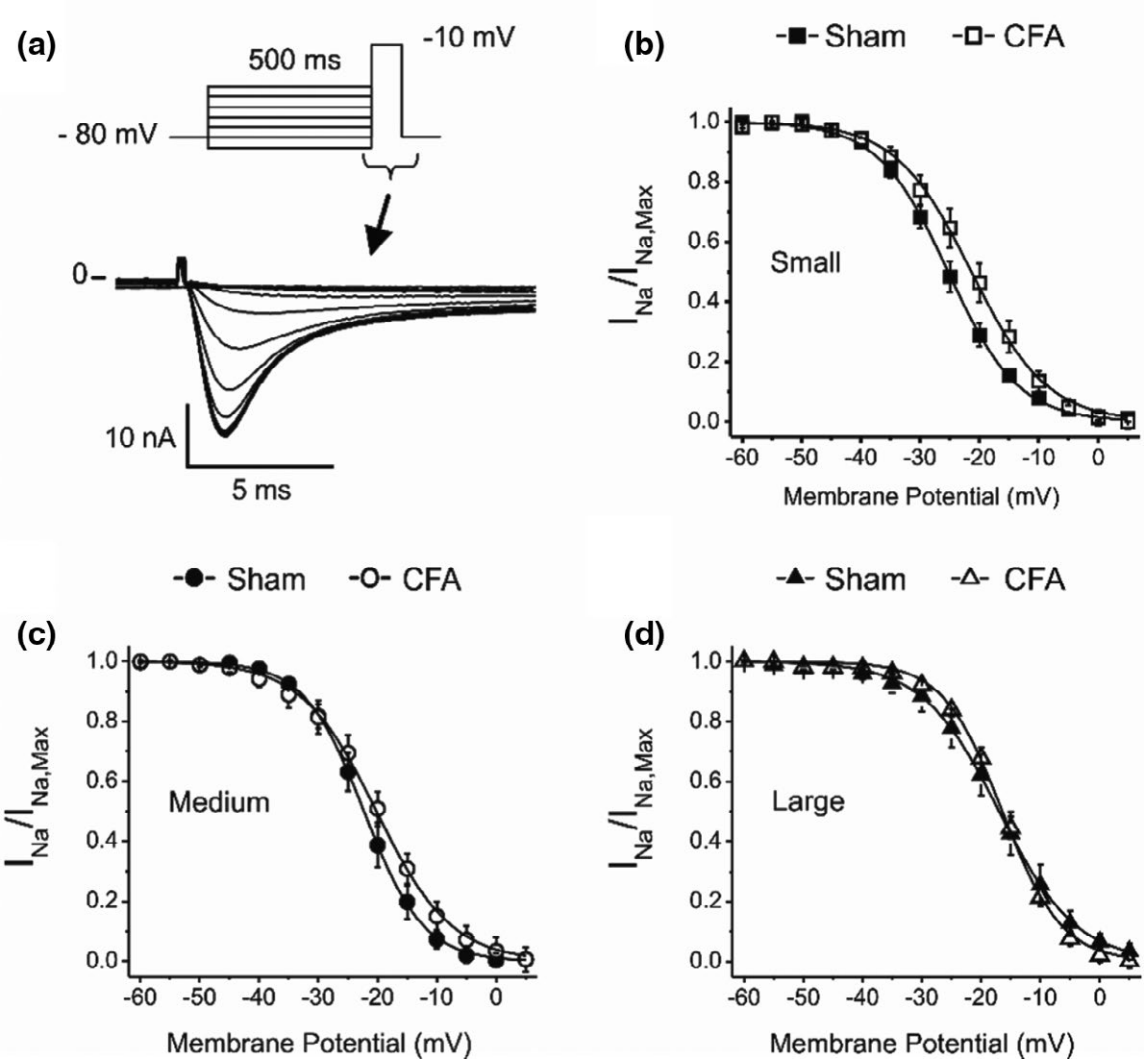
Complete Freund's adjuvant does not change the availability of sodium channels in medium and large neurons. (a) Representative recordings of I_Na_ following a 500 ms conditioning pulse to various voltages from a holding potential of −80 mV (inset). (b–d) Relative current as a function of the conditioning membrane potential. Data ± SEM fitted to a standard Boltzmann distribution (see Section 2)

We next tested if the increase in I_Na_ amplitude was due to voltage‐dependent changes in the availability of the channels that would be indicative of a phosphorylation process or a mixed population of channels. Steady‐state inactivation determines the maximum amplitude of I_Na_ by modulating the number of channels available for opening at various resting membrane potentials. Figure 6 shows that I_Na_ half‐inactivation voltage (V_h0.5_) was similar in small (−25.3 ± 0.1 mV) and medium neurons (−22.3 ± 01 mV) from Sham animals, but it was more positive in large cells (−16.4 ± 0.2 mV). This further suggests that a different population of Na^+^ channels may contribute to I_Na_ in large neurons. CFA did not alter the availability of sodium channels with V_0.5_ of −20.2 ± 0.2 and −16.4 ± 0.2 mV in medium and large cells, respectively. In contrast, CFA shifted V_h0.5_ to −21.3 ± 0.2 mV (*p *< 0.05, *F*‐test) in small cells which could explain the increase in V_max_ shown in Figure 2g.

**FIGURE 7:**
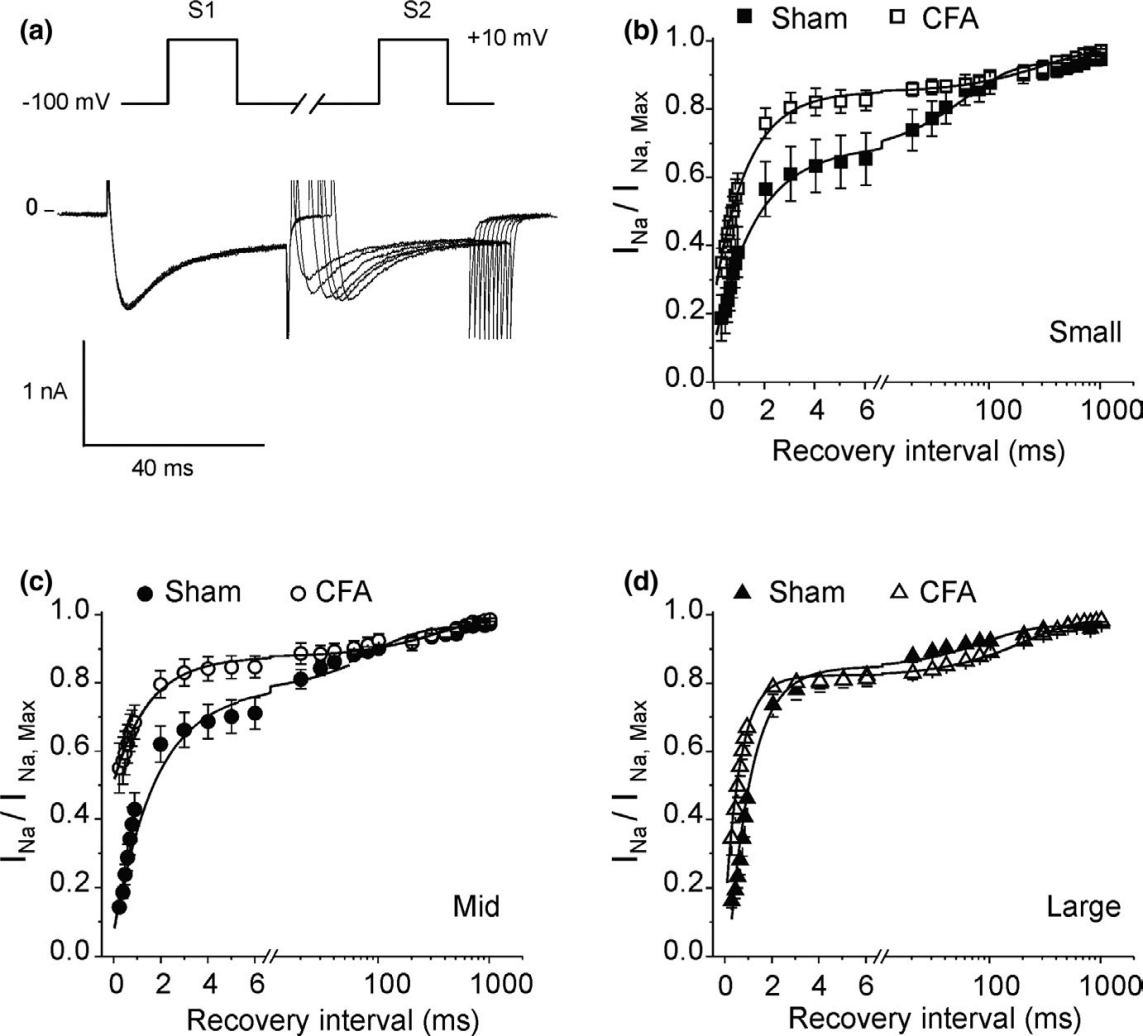
Complete Freund's adjuvant abolishes the difference in recovery from inactivation between small, medium, and large neurons. (a) Representative current recordings during a dual pulse protocol with varying recovery intervals. (b–d) Fraction of channels recovered during the interpulse (recovery) interval for each cell type. Data were fitted to a sum of two exponentials (solid line). Data + SEM (*n* = 7–9 for each cell type, six animals)

Selective alterations in the firing properties of sensory neurons will tip the balance of the signal toward pain. Neurotransmission and the level of pain both rely on a fine balance between excitation and inhibition of each neuronal cell type (Guo & Hu, 2014). A key determinant of this balance is the maximum rate at which each neuronal cell type can trigger APs. This firing rate is in large part controlled by the time needed for I_Na_ to recover from inactivation upon return to resting membrane potential and channels to return closed states from where they are again available to open upon stimulation. This interval creates a refractory period during which it is impossible to trigger an AP and imposes a limit on the firing frequency of sensory neurons. We, therefore, tested for differences in recovery time of I_Na_ between neuronal cell types. In Sham animals, peak I_Na_ recovered 82 ± 3% of its amplitude within 6 ms in large neurons, while recovery from inactivation was slower and reached only 65 ± 7% and 70 ± 5% for the same interval in small and medium cells, respectively (Figure 7). CFA accelerated recovery in small and medium‐sized neurons but had no effect in large cells such that the recovery rate became similar in all cell types. CFA abolished differences in the refractory period between each cell type to favor nociceptive signals. Table 2 shows that the acceleration of recovery by CFA was primarily due to an increased rate of recovery rather than a change in the amplitude of the slow and fast components.

**TABLE 2:** Parameters from recovery from inactivation fit to a sum of two exponentials. A and τ: percent and rate constant associated with the fast (F) or slow (S) component of recovery. Statistical significance indicates a difference between fit to data: *p < 0.05 (Sham vs. CFA), ^†^ *p *< 0.05 (vs. large) in Sham condition, ^‡^ *p *< 0.01 (small vs. large) in CFA condition. Number of cells: 7–9 per cell type from six animals

	Sham				CFA			
	A_F_ (%)	τ_F_ (ms)	A_S_ (%)	τ_S_ (ms)	A_F_ (%)	τ_F_ (ms)	A_S_ (%)	τ_S_ (ms)
Small*^,†,‡^	67	1.6 ± 0.53	33	62 ± 18	83	1.31 ± 0.15	17	299 ± 98
Mid*^,†^	78	1.24 ± 0.21	22	72 ± 36	87	0.71 ± 0.05	13	243 ± 122
Large	88	0.96 ± 0.06	12	89 ± 31	80	0.65 ± 0.08	20	205 ± 79

**FIGURE 8:**
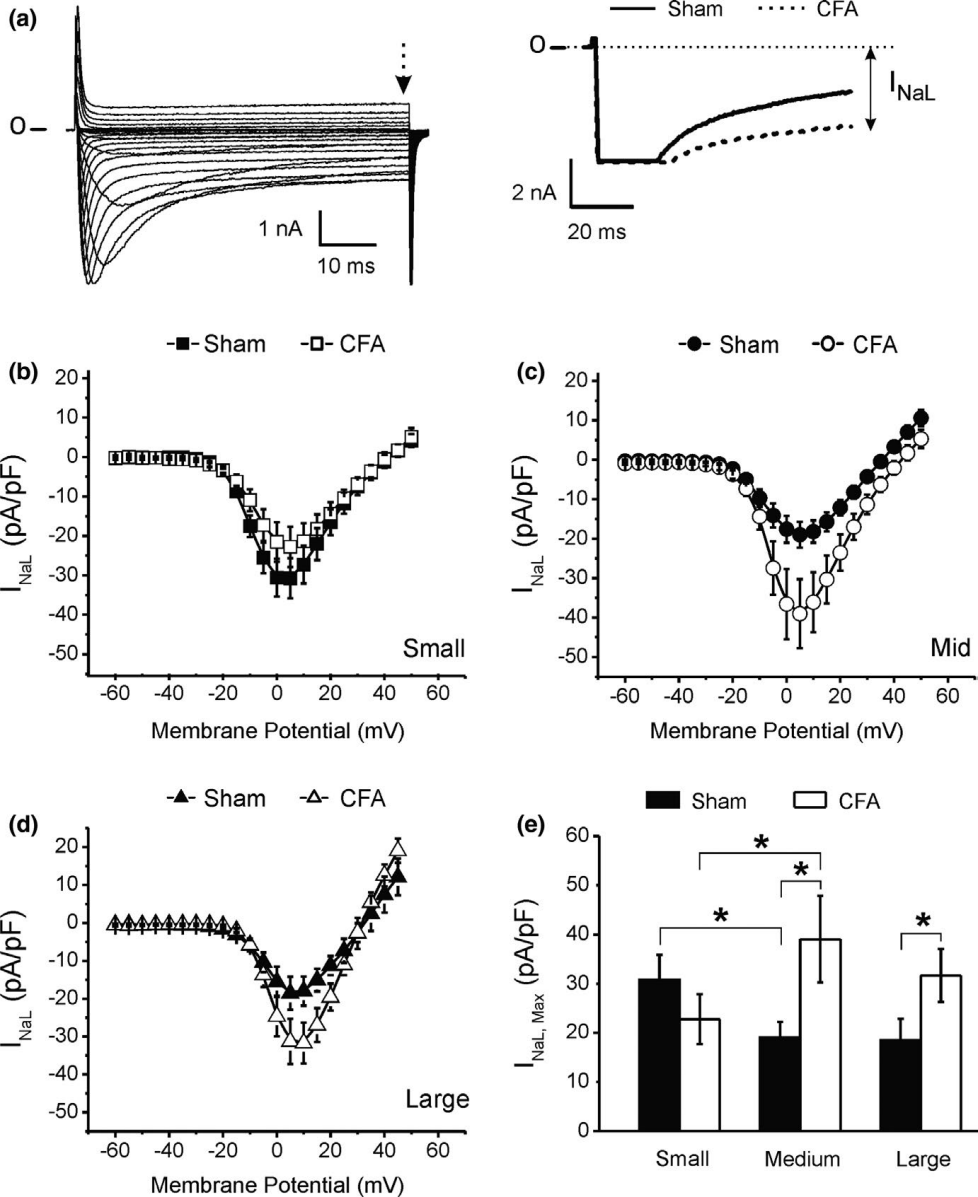
Complete Freund's adjuvant (CFA) increased the amplitude of the TTX‐r late Na^+^ current density (I_NaL_) only in medium and large neurons (a) Representative current recordings during a series of 60 ms depolarizing pulses from a holding potential of −60 mV. Dotted arrow indicates the time of the measurement of the sustained (late) component of I_Na_. Panel on the right indicates how the amplitude of I_NaL_ was measured (double arrow). (b–d) I_NaL_ current‐voltage relationship following CFA treatment (data ± SEM). (e) Maximum I_NaL_ amplitude in the conditions tested. Statistics: *p < 0.05. Number of cells = 9–12 from six different animals. Data ± SEM

Another determinant of the refractory period and firing frequency in neurons is the duration of the action potential (APD). Longer AP will inactivate more sodium channels and slow the recovery of I_Na_, thus prolonging the refractory period. A key element modulating APD is the amplitude of the sustained component of I_Na_. This late current (I_NaL_) will maintain a depolarizing force that ultimately delays the repolarization of the cells and influence the threshold for triggering APs (Baker & Bostock, 1997; Cummins et al., 1999; Cummins & Waxman, 1997). Given the AP prolongation observed in large neurons after CFA treatment, we wondered if I_NaL_ was differentially modulated between each neuronal cell type. We next measured I_NaL_ at the end of a 60 ms depolarizing pulse (Figure 8).

In Sham animals, I_NaL_ amplitude was largest in small neurons but similar between medium and large‐sized cells (Figure 8e). CFA had no significant effect on I_NaL_ in small cells (Figure 8b) but selectively increased I_NaL_ in medium and large neurons (Figure 8c,d), thereby reversing the amplitude distribution found in Sham animals. The increase in I_NaL_ in large neurons may contribute to the longer APD during chronic inflammation but did not seem sufficient to generate the depolarizing force needed to prolong APD in medium‐sized cells. This suggests that CFA modulated other ionic currents to compensate for the depolarizing effect of I_NaL_ on the action potential of medium cells.

Among the potential compensatory mechanisms are a reduction of the calcium current density I_Ca_ or an increase in potassium outward current density (I_K_). In Figure 3, we showed that I_K_ decreased in medium‐sized neurons despite no changes in APD during CFA‐induced inflammation (Figure 2). This indicates that the decrease in I_K_ combined by the increase in I_NaL_ (Figure 8) had to be compensated by the reduction of another inward current. We, therefore, measured the amplitude of I_Ca_.

**FIGURE 9:**
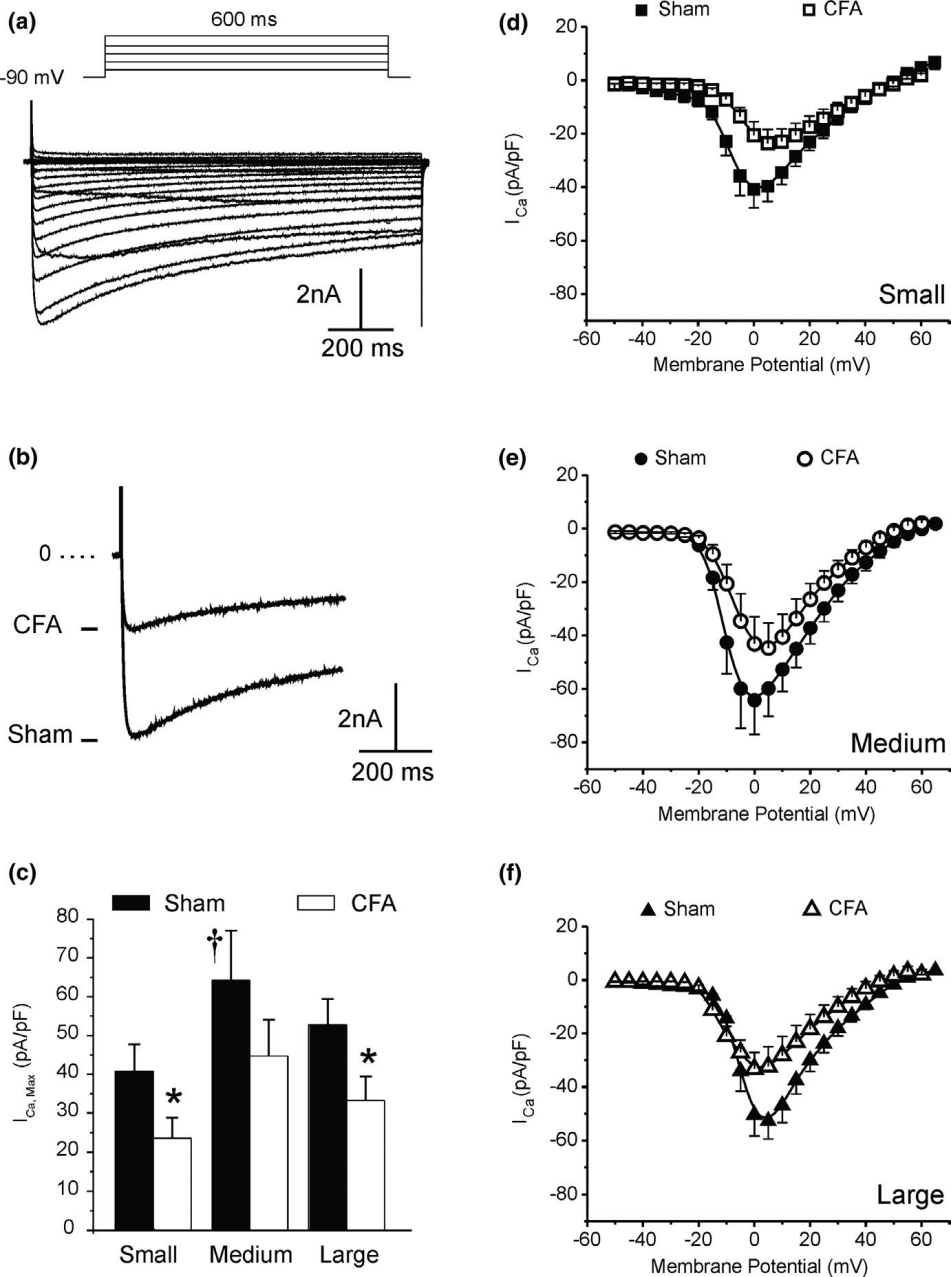
Complete Freund's adjuvant (CFA) reduces the slow calcium current density in DRG neurons. (a and b) Representative current recordings from a large neuron in Sham animal (a) and following exposure to Freund's adjuvant (b). (c) Maximum amplitude (±*SD*) of I_CaL_ in small, medium, and large cells in Sham and CFA exposed rats. (d–e) Current–voltage relationship for each cell type and conditions. Data ± SEM was obtained from 7 to 13 cells from six animals. Statistical significance: *p < 0.05 versus Sham, ^†^ *p *< 0.05 versus small

**FIGURE 10:**
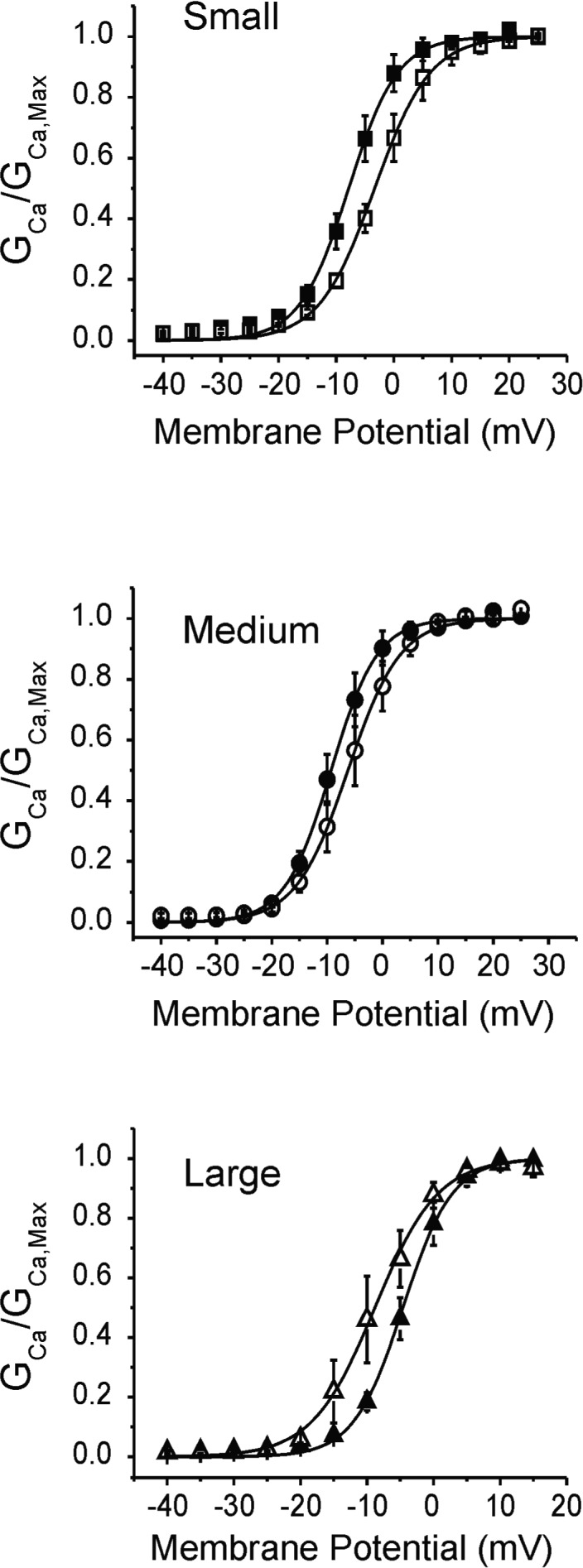
Complete Freund's adjuvant displaced the activation voltage of ICaL. Activation curves obtained from the ratio of conductance at each stimulating potential to maximum conductance calculated from the linear portion of the I/V relationship presented in Figure 9. Data ± SEM

We detected two types of calcium currents, namely type T (fast) and type L (slow). However, I_CaT_ was present in <10% of the cells studied, we, therefore, focused on I_CaL_ which could be readily recorded in all cell types. Figure 9 shows that CFA reduced I_CaL_ amplitude by 42% and 37% in small and large neurons, respectively. Despite a trend toward a reduction in I_CaL_ in medium‐sized cells, the difference between Sham and CFA values (30%) did not reach a significance level in our experiments. Biophysical analysis revealed a small depolarizing shift in mid‐activation potentials of small‐ and medium‐sized neurons. Interestingly, a hyperpolarizing shift was observed in large neurons (Figure 10; Table 3). We did not investigate further the reasons for this shift in our conditions since the current kinetics was not altered. However, it is well‐known that variations in basal calcium and phosphorylation by protein kinases (A and C) modulate the voltage dependence of activation and inactivation of I_CaL_. We speculate that the variations in maximum current are due to differences in the activity of kinases and intracellular calcium levels in each cell type.

**TABLE 3:** Biophysical parameters for the activation of I_CaL_ obtained from the I/V relationship (Figure 9) and Boltzmann distribution fit to conductance data (Figure 10). Statistical significance (ANOVA vs. Sham values): *p < 0.05, **p < 0.01, ***p < 0.001

	Sham		CFA	
	I_Ca,Max_ (pA/pF)	V_1/2_ activation (mV)	I_Ca,Max_ (pA/pF)	V_1/2_ activation (mV)
Small	41 ± 7	7.8 ± 0.2	24 ± 5*	3.6 ± 0.2***
Medium	64 ± 13	9.3 ± 0.1	45 ± 9	6.2 ± 0.2*
Large	53 ± 7	5.6 ± 0.2	33 ± 6*	8.8 ± 0.2**

## 4 DISCUSSION

### 4.1 Modulation of the action potential in large sensory Aβ fibers during inflammation

Our data indicate that large sensory neurons display the shortest action potential duration in Sham condition, compared to small and medium sized. Those myelinated Aβ fibers normally convey the tactile and proprioceptive inputs and are generally considered to be non‐nociceptive under physiological conditions. Importantly, we also found that CFA treatment prolonged APD solely in large neurons. A well‐known consequence of such change in the electrophysiological properties of large myelinated Aβ afferent fibers is the development of mechanical allodynia in chronic pain states (Tsuda, 2019; Zhu et al., 2012). This result is consistent with previous observations of allodynia in this CFA model and may be explained by the hyperactivity of Aβ fibers caused by APD prolongation (Belkouch et al., 2014; Hoseini et al., 2006; Zhu et al., 2012).

There is now considerable evidence supporting the idea that the hyperexcitability of Aβ afferent fibers plays an important role in the pathophysiology of chronic pain (Frank et al., 2019). Hyperalgesic states induced by peripheral injury or inflammation, indeed, enhance the synaptic connections between Aβ afferent fibers and nociceptive projection neurons (Kuner, 2010). Our finding of a longer APD in large neurons is consistent with this synaptic plasticity and spinal reorganization. The Aβ afferent fibers are expected to become more excitable during chronic inflammation and shift the balance of neuronal activity toward pronociceptive (pain‐promoting) signaling. This shift in the balance of activity between the various neuronal cell subtypes may represent the primary signaling mechanism driving the spinal nociceptive processing and pain sensation. Our results highlight the important role of large sensory neurons in this process and indicate that their hyperactivity is one of the mechanisms involved in nociceptive neurotransmission during CFA‐induced inflammation.

Our results also revealed that these changes in AP waveforms are the result of a complex interaction between I_K_, I_Na_, and I_Cal_.

### 4.2 Contribution of I_K_

Voltage‐gated K^+^ channels are critical in controlling neuronal excitability and their dysfunction in sensory neurons may result in the persistent pain state (Zemel et al., 2018). Here, we found that I_K_ amplitude is similar in small and medium‐sized neurons but significantly smaller (63 ± 7%) in large neurons from Sham animals. Interestingly, these differences occur because the contribution of I_A_ is larger in nociceptive neurons, accounting for 40% to 50% of I_K_ in small and medium cells, but only 20% in large neurons. CFA did not significantly increase I_K_ amplitude at 20 mV in large or small neurons but importantly decreased I_K_ by one‐third in medium‐sized cells. Nonetheless, the fraction of I_A_ contributing to I_K_ remained the same in all neuronal cell types during inflammation. Reductions in I_A_ currents have also been proposed to underlie neuronal hyperexcitability in other inflammatory pain models. For instance, antigen‐induced arthritis (AIA) used to study rheumatoid arthritis enhances the excitability of joint sensory neurons through a reduction in the density of I_A_ currents (Qu & Caterina, 2016). Likewise, joint inflammation induced by CFA leads to a decrease in both I_A_ magnitude and Kv1.4 expression in small nociceptive trigeminal ganglion neurons (Takeda et al., 2006, 2008). This downregulation in A‐type K^+^ channel gene expression and the reductions in I_A_ currents have also been reported in other chronic pain conditions, such as spinal nerve ligation and diabetic models of neuropathic pain (Cao et al., 2010; Chien et al., 2007). The underlying mechanisms by which peripheral inflammation decreases the expression and activity of I_A_ currents need further investigation. However, some inflammatory mediators, such as the cytokine interleukin 1β and glial cell line‐derived neurotrophic factor (GDNF) have already been demonstrated to reduce I_A_ in both small and medium nociceptive neurons (Stemkowski et al., 2015; Takeda et al., 2010). Because I_A_ is significantly larger in small and medium‐sized neurons, it is, therefore, a potential target to specifically modulate APD in nociceptive neurons with minimal impact on tactile and proprioceptive inputs. Our data, therefore, provide an additional mechanism to explain the beneficial effects of IA blockers. Overall, our results thus suggest that the manipulation of I_A_ more specifically affects neuronal excitability and the subsequent transmission of the nociceptive message.

Since the suppression of I_A_ leads to neuronal hyperexcitability, I_A_ activation might reduce spike duration and frequency as well as an increase in spike threshold, thus leading to the specific inactivation of small and medium nociceptive neurons versus large ones and then the reduction in inflammatory pain. In that sense, the activation of the neuromedin U type 1 receptor (NMUR1) and serotonin type‐1D receptor (5‐HT_1D_) have been found to selectively increase I_A_ and then contribute to the decrease in neuronal excitability (Zhang et al., 2012; Zhao et al., 2016). In addition, the cobrotoxin isolated from the venom of Taiwan cobra Naja naja atra, which produces strong analgesic effects via the activation of the muscarinic M3 receptor (M3R) also decreases the neuronal hyperexcitability following the activation of I_A_ currents (Guo et al., 2013). Finally, the nonsteroidal anti‐inflammatory drug (NSAID), diclofenac commonly used to control pain and inflammation also attenuates bone cancer pain by enhancing I_A_ currents (Duan et al., 2012).

### 4.3 Contribution of I_Na_

Previous findings showed the important role of voltage‐gated sodium channels in the excitability of sensory neurons. Among them, four (Na_v_1.3, Na_v_1.7, Na_v_1.8, Na_v_1.9) have been described as major contributors in the generation and propagation of neuronal action potentials (Bennett et al., 2019; Waxman & Zamponi, 2014). Here, we did not observe significant differences in peak I_Na_ amplitude between small, medium, and large neurons in sham DRGs. However, mid‐activation voltage was 6.6 mV more positive in large neurons versus small ones. CFA increased I_Na_ in large neurons and altered the activation gating only in small cells. These changes in I_Na_ did not have a significant impact on the rising phase of the action potential since V_max_ and the maximum amplitude of the action potential were not altered. Nonetheless, the more depolarized activation voltage of I_Na_ in large versus small neurons raises the possibility that different populations of TTX‐r sodium channels exist in each cell type.

Analysis of Figures 5c–e and 6 further suggests that a different population of Na^+^ channels is expressed in large neurons. From Ohms law, maximum peak current I_Na,Max_ = G_Na,max_·ΔV where G_Na,max_ tells us how many channels are open and available (Figure 5d). Conductance on the other hand (Figure 5e) can be viewed as the product N·P_o_·g_Na_ where N = max nb of channel, Po = opening probability, and g_Na_ = unitary conductance of each channel. Under this formulation I_Na_= N·P_o_·g_Na_·ΔV, where ΔV corresponds to the driving force on the Na^+^ ion.

Once all channels are open (P_o_ = 1), G_Na,max_ = N·g_Na_ and conductance is directly proportional to the number of channels expressed and their single‐channel conductance.

In the Hodgkin–Huxley model, G_Na_ can also be written as G_Na_ = h·m^3^·gNa, where h is the maximum availability of the channels (inactivation) and corresponds to N when the holding membrane potential is fixed (as in our I/V protocol). Parameter m^3^ = opening probability (P_o_) and is given by the voltage dependence of activation. Depolarization or hyperpolarization of P_o_ will change the voltage at which I_Na_ reaches its maximum but will not have any effect on G_Na, Max_. Our data (Figure 5c) indicate that CFA did not change I_Na_ activation (m^3^) but increased I_Na, Max_ by 53% ± 2% and G_Na,max_ by 40% ± 4% (Figure 5d,e).

The number of channels contributing to I_Na_ will also depend on their availability (inactivation). However, we did not find any changes in I_Na_ inactivation (h) in large neurons. Since no changes in I_Na_ biophysical properties can explain the increase in I_Na,max_ and G_Na,max_, we can reasonably conclude that CFA increased the number of channels contributing to I_Na_. In support, we previously reported that chronic peripheral inflammation increased the expression of NaV1.8 in DRG neurons (Belkouch et al., 2014).

Moreover, our observation of mid‐inactivation potentials more positive in large cells versus small and medium neurons in Sham animals combined with the fact that CFA only altered the availability of the channels in small cells, indicating a highly heterogeneous distribution of Na^+^ channel isotypes in DRG neurons. As stated earlier, the balance of excitatory and inhibitory inputs coming from nociceptive and anti‐nociceptive neurons will determine the level of pain perceived by the brain. Excitability and the rate at which each neuron can trigger action potentials (firing rate) determine the activity of each neuronal subtype. Excitability is mostly determined by the availability of sodium channels and the voltage threshold for triggering an action potential. We found that the AP threshold was more negative and closer to the resting membrane potential in large myelinated tactile Aβ afferent fibers versus small and medium nociceptive neurons. Therefore, the activation of nociceptive neurons requires a larger stimulus than anti‐nociceptive cells. This suggests that the threshold for pain in Sham animals is for the most part determined by the activity of large anti‐nociceptive neurons. CFA had no effect on the AP threshold in DRG neurons, suggesting that the small changes in I_Na_ activation and steady‐state inactivation we observed did not directly alter neuronal excitability. Therefore, the perceived level of pain linked to CFA inflammation is not caused solely by changes in the excitability of nociceptive neurons. This lends further support to I_A_ and APD as a major determinant of chronic pain.

### 4.4 CFA and the refractory period

The time needed for I_Na_ to recover after an AP creates a refractory period limiting neuronal firing rate. Our data show that sodium channels recover much faster in large neurons compared to small and medium cells in Sham animals. Therefore, large neurons are likely to have a higher firing rate than small or medium cells. This observation combined with the more hyperpolarized AP threshold in large cells further support the hypothesis that large neurons act as dampers to set the threshold for pain under physiological condition.

An important finding was that CFA selectively accelerated recovery from inactivation in small and medium cells only. This faster recovery is likely to selectively increase the maximum firing frequency and the excitability of these nociceptive neurons. CFA had no effects on I_Na_ recovery in large neurons but prolonged their APD. Longer AP durations are known to increase the refractory period. These results suggest that CFA reduces the threshold for pain by concomitantly enhancing the activity of nociceptive neurons and reducing the inhibitory action of large neurons. However, it would be difficult to determine precisely how these changes in the absolute refractory period would impact AP firing. Indeed, firing frequency depends on several factors, such as the rate of repolarization and the threshold potentials, which are also dependent on other factors, like I_K_ and I_Ca_. Nonetheless, our results suggest that chronic peripheral inflammation will increase the maximum firing frequency in C and Aδ nociceptive fibers but will have no effect on the excitability of non‐nociceptive neurons. One logical conclusion is that part of the pain signaling involves the selective reduction of the refractory period in nociceptive neurons. Previous studies proposed that pro‐inflammatory cytokines involved in the generation of pain‐sensitized primary afferent nociceptors by increasing the amplitude of I_Na_ (Dib‐Hajj et al., 2010; Gold, 1999; Gold et al., 1996). Our data suggest a more complex mechanism in which chronic inflammation is shifting the balance of neuronal activity in DRG toward nociception by dual but opposite actions on C, Aδ, and Aβ fibers.

### 4.5 Contribution of I_NaL_

Action potential duration is in part determined by the amplitude of I_NaL_. Our data indicate that I_NaL_ is largest in small cells but comparable between medium and large neurons from Sham animals. This finding is consistent with previous studies showing that slowly inactivating Na_V_1.9 channels are selectively expressed in nociceptive neurons where they regulate the AP threshold (Baker et al., 2003; Herzog et al., 2001). Interestingly, CFA increased I_NaL_ amplitude only in medium and large cells. Analysis of I_NaL_ expressed as a fraction of the peak current revealed a proportional change between I_NaL_ and I_Na_ in large neurons, consistent with an effect on a single Na_V_ isotype and our previous findings of an increased expression of Na_V_1.8 during inflammation (Belkouch et al., 2014). In contrast, CFA increased I_NaL_ but had no effects on peak I_Na_ in medium neurons. This suggests that inflammation triggered the expression of a different sodium channel isotype having a large ratio of late to peak sodium current in medium‐sized cells. Based on previous studies, one likely candidate is Na_V_1.9 (Baker et al., 2003; Herzog et al., 2001). Alternatively, CFA may differentially alter the intracellular cascade modulating the late activity of the channels in each cell type. This last hypothesis is supported by observations indicating that Na^+^ channels Na_V_1.8 and Na_V_1.9 react differently to phosphorylation by kinases (Scheuer, 2011; Smith & Goldin, 2000) or G‐proteins (Kakimura et al., 2010; Ostman et al., 2008). For example, the surface expression of the TTX‐r isoform Na_V_1.8 is enhanced upon phosphorylation by PKA, PKC or P38‐mitogen‐activated kinase during inflammation, while phosphorylation is associated with G‐protein activation selectively enhances Na_V_1.9 late currents. Further experiments are needed to delineate between the expression or phosphorylation mechanisms.

### 4.6 Contribution of I_CaL_

Voltage‐gated calcium channels (VGGCs) exert a critical role in neuronal functions (Gribkoff, 2006). By controlling Ca^2+^ entry, they notably drive neurotransmitter release at central terminals of sensory neurons in the spinal dorsal horn and regulate afferent fiber excitability. VGCCs are classified into five subfamilies L‐, P/Q‐, N‐, R‐ subtypes (high‐voltage activated) and T‐type channels (low‐voltage activated), based on a combination of biophysical and pharmacological properties (Bourinet et al., 2016; Li et al., 2019). To date, each calcium channel subtype has been implicated in the development and persistence of several pain conditions, with N‐ (Cav2.2) and T‐type (Cav3.2) VGCCs being of particular interest as pain targets (Patel et al., 2018; Zamponi et al., 2015). Accordingly, pharmacological inhibition of these channels by selective blockers was found to produce analgesia. For instance, the T‐type calcium channel inhibitor ethosuximide attenuates the nociceptive behaviors associated with painful neuropathies, such as traumatic nerve injury, diabetic neuropathy, and chemotherapy‐induced peripheral neuropathy (François et al., 2014). Likewise, the FDA‐approved Ziconotide (Prialt), Gabapentin (Neurontin), and Pregabalin (Lyrica), acting as CaV2.2 blockers reduce both hyperalgesia and allodynia in chronic pain states, through the spinal presynaptic inhibition of neurotransmitter release from primary afferent neurons (Bourinet & Zamponi, 2017; Patel et al., 2018).

Our experiments indicate that I_CaL_ is slightly larger in medium cells versus small and large neurons. This may compensate for the smaller I_NaL_ and provide the depolarizing force needed to maintain the duration of the AP in medium neurons. Furthermore, we observed that I_CaT_ accounted for less than 10% of the total calcium current, a result consistent with previous studies showing that L‐ and N‐type currents contribute to most of I_Ca_ in sensory neurons (McCallum et al., 2011). In CFA condition, the amplitude of I_CaL_ was similarly reduced in each cell type. This result is consistent with previous findings showing the loss of I_Ca_ in nociceptive neurons after peripheral axotomy or inflammation (Lu et al., 2010; McCallum et al., 2011). Mechanistically, this decrease in Ca^2+^ influx via VGCCs in DRG cell body will reduce the opening of Ca^2+^‐dependent K^+^ channels, and‐ as a consequence‐depolarize the resting membrane potential of neurons closer to the action potential threshold to enhance nociception (Berkefeld et al., 2006; Hogan et al., 2008; Scholz et al., 1998). This effect might be exacerbated by the small depolarizing shift in the mid‐activation potential we observed in small‐ and medium‐sized neurons which will tend to further reduce the activity of I_CaL_. Interestingly, a hyperpolarizing shift of mid‐activation potential was observed in large neurons. Given the implication of large neurons in mechanical sensation, we speculate that this shift in activation may promote allodynia since the current will activate at potentials closer to the RMP. Changes in the expression and axonal transport of calcium channels to central terminals in the spinal dorsal horn are thought to be a contributing factor to the reduction of I_Ca_ within the neuronal soma (Leo et al., 2017; Lu et al., 2010; Murali et al., 2015). Together, these results provide additional supports for the contribution of VGCCs in regulating the hypersensitivity associated with persistent inflammation.

### 4.7 Limitations

In this study, we separated neurons based on their size. Despite this selection, it remains possible that a small overlap between non‐peptidergic C‐fiber neurons and peptidergic C, Aβ, and Aδ nociceptors might exist in our measurements. Although this may lead to slight differences in the interpretation of the mechanisms (mechanical vs. thermal) by which pain is transmitted, this will not change our overall results showing that CFA induces changes in the balance of the activity of nociceptive and anti‐nociceptive neurons within the DRGs.

In summary, our study shows that the pain signal during inflammation is not solely linked to the excitability of nociceptive neurons but results from complex and different modulations of the AP properties and ion channels in at least three types of neurons. We observed a selective prolongation of the action potential in tactile Aβ afferent fibers and a decrease in the refractory period of nociceptive Aδ and C fibers. These changes will concur in shifting the balance of anti‐nociceptive to nociceptive signal, thus favoring the development of a hyperalgesic state. Our results also suggest that targeting I_A_ in small and medium‐sized neurons should be an efficient way to shift the balance toward anti‐nociception during inflammation. Indeed, since I_A_ contributes minimally to AP in large neurons, its activation will selectively reduce APD and spike frequency in nociceptive neurons. This represents a new avenue for treating pain linked to chronic inflammation.

## COMPETING INTERESTS

The authors declare that they have no known competing financial interests or personal relationships which have, or could be perceived to have, influenced the work reported in this article.

## AUTHOR CONTRIBUTIONS

PS and RD conceptualized and designed the study. MB performed the electrophysiological experiments and analyzed data. MAD performed the in vivo experiments and analyzed data. All authors edited and revised the manuscript and approved the final version of the manuscript.

## Supporting information



Fig S1

## Data Availability

The data that support the findings of this study are available from the corresponding author upon reasonable request.
