# Supplementary material for: The neuronal potassium current IA is a potential target for pain during chronic inflammation
Source: Physiol Rep. 2021 Aug 17;9(16):e14975. doi: 10.14814/phy2.14975 (PMC8371350; doi:10.14814/phy2.14975)
Supplement: Supplementary file 1 — Fig S1 [file PHY2-9-e14975-s001.docx]

**
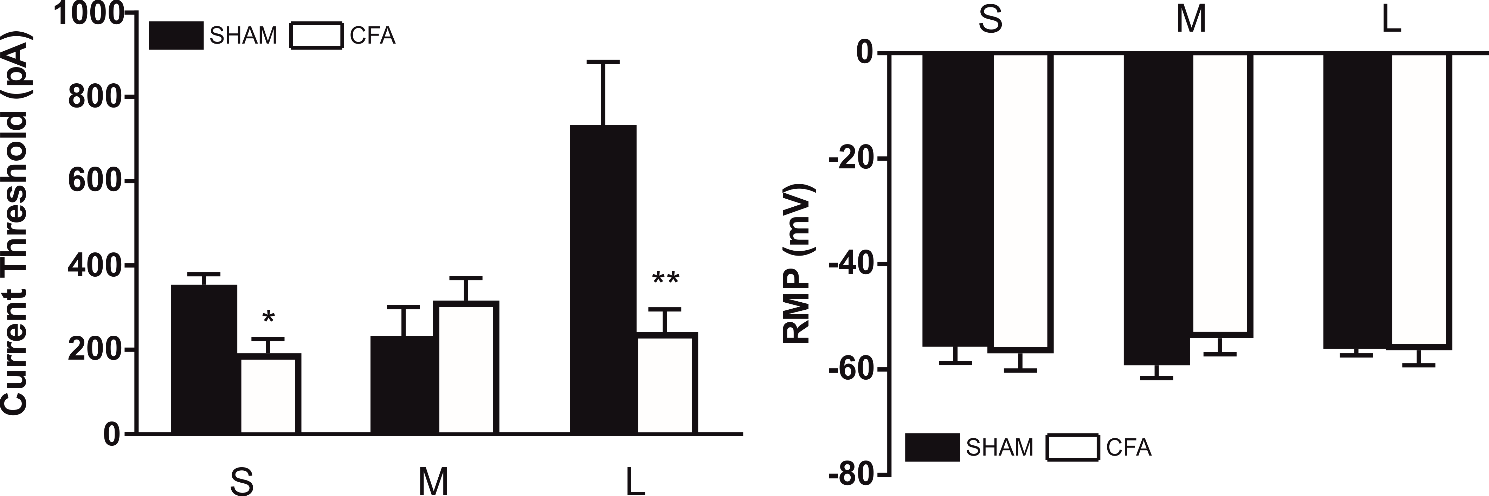
**

**Figure supplemental S1.** Threshold current needed to trigger an action potential (left panel) and resting membrane potential recorded after contact with the intracellular milieu. (Data ± SEM, n=10 from 4 animals).
